# Shortcomings of adherence counselling provided to caregivers of children receiving antiretroviral therapy in rural South Africa

**DOI:** 10.1080/09540121.2016.1176675

**Published:** 2016-07-08

**Authors:** Bronwyne Coetzee, Ashraf Kagee, Ruth Bland

**Affiliations:** ^a^ Department of Psychology, Stellenbosch University, Stellenbosch, South Africa; ^b^ Africa Centre for Population Health, KwaZulu-Natal, South Africa; ^c^ Institute of Health and Wellbeing and Royal Hospital for Sick Children, University of Glasgow, Glasgow, UK; ^d^ School of Public Health, Faculty of Health Sciences, University of Witwatersrand, Johannesburg, South Africa

**Keywords:** Antiretroviral therapy, adherence, counselling, paediatric

## Abstract

In order to achieve optimal benefits of antiretroviral therapy (ART), caregivers of children receiving ART are required to attend routine clinic visits monthly and administer medication to the child as prescribed. Yet, the level of adherence to these behaviours varies considerably in many settings. As a way to achieve optimal adherence in rural KwaZulu-Natal, caregivers are required to attend routine counselling sessions at HIV treatment clinics that are centred on imparting information, motivation, and behavioural skills related to medication administration. According to the information-motivation-behavioural skills model, information related to adherence, motivation, and behavioural skills are necessary and fundamental determinants of adherence to ART. The purpose of the study was to observe and document the content of adherence counselling sessions that caregivers attending rural clinics in KwaZulu Natal receive. We observed 25 adherence counselling sessions, which lasted on average 8.1 minutes. Counselling typically consisted of counsellors recording patient attendance, reporting CD4 count and viral load results to caregivers, emphasising dose times, and asking caregivers to name their medications and dosage amounts. Patients were seldom asked to demonstrate how they measure the medication. They were also not probed for problems regarding treatment, even when an unsuppressed VL was reported to a caregiver. This paper calls attention to the sub-optimal level of counselling provided to patients on ART and the urgent need to standardise and improve the training, support, and debriefing provided to counsellors.

## Introduction and background

In order to achieve the optimal benefits of antiretroviral therapy (ART), such as an increased CD4 count and a suppressed viral load (VL), excellent adherence (>95%) to ART is required (Bangsberg et al., 2001, 2003; Paterson et al., 2000). Yet, medication adherence has complex behavioural, cognitive, and affective antecedents and requires large amounts of motivation and support (Rapoff, 2010). In the case of young children, adherence to ART is further complicated as the responsibility for treatment lies with a parent or caregiver, and not directly with the child.

The most robust model for conceptualising ART adherence is the information-motivation-behavioural (IMB) skills model (Fisher, Fisher, Amico, & Harman, 2006). In the case of paediatric adherence to ART, information related to adherence requires that caregivers have accurate knowledge about the illness that is relevant to support adherence. Caregivers need to be sufficiently motivated to adhere and be able to negotiate the individual and structural barriers they encounter, such as inadequate transport to clinics and long waiting times (Coetzee, Kagee, & Vermeulen, 2011). Behavioural skills entail the moment-to-moment physical and logistical skills required to administer medication to the child correctly and punctually.

One of the ways in which adherence to ART is supported by the South African ART programme is through patients’ monthly clinic visits to an adherence counsellor. South Africa has the largest ART programme worldwide (Joint United Nations Programme on HIV/AIDS, 2014). Thus, in an attempt to manage the ever-expanding ART programme in the context of insufficient health workers, the South African Department of Health has adopted a task-shifting approach to manage the sheer numbers of ART users. At present, ART treatment is predominantly nurse- and counsellor-led (McPake & Mensah, 2008).

Adherence counsellors in the South African healthcare system are lay personnel (i.e., do not typically hold professional/paraprofessional qualifications) who provide health-seeking individuals with health counselling and testing (HCT) (Dewing et al., 2015; Petersen, Fairall, Egbe, & Bhana, 2014). The length of training adherence counsellors receive varies between three days (Coetzee, Kagee, & Bland, 2015) and one year (Remien et al., 2013). Following their training, adherence counsellors are responsible for HCT, patient education, counselling, adherence support and community outreach, often without further training or regular supervision (Dewing et al., 2012; Petersen et al., 2014; Remien et al., 2013).

In previous work, we conducted a qualitative study exploring the barriers and facilitators to adherence to ART amongst children and their caregivers living in rural KZN (Coetzee et al., 2015). Interviews with doctors and nurses in the treatment and care of children on ART in the study area suggested that the adherence counselling sessions received by caregivers during their monthly visits to the clinics were poorly conducted, as a result of the rushed training that counsellors received and the lack of counsellor supervision and debriefing. As such, doctors and nurses considered counsellors unable to provide adequate adherence support.

Counsellors are well positioned in the healthcare system to provide caregivers with the information, motivation and skills necessary to adhere optimally to ART. However, little is known about the content of routine adherence counselling sessions, and to what extent lay counsellors are indeed able to provide adherence support. In this study, we observed and documented the adherence counselling sessions that primary caregivers to children younger than five years received when attending a monthly clinic visit.

## Methods

### Setting

The study was conducted at two primary healthcare clinics operating within the Hlabisa sub-district, in Northern KwaZulu Natal, which provide HIV treatment as part of the Department of Health Hlabisa Treatment and Care Programme (HTACP) (Houlihan et al., 2011; Janssen, Ndirangu, Newell, & Bland, 2010). The Africa Centre for Population Studies (www.africacentre.com) has supported the HTACP since its inception in 2004 (Houlihan et al., 2011; Janssen et al., 2010).

### Sample

We purposively recruited 33 caregiver–child dyads into a larger qualitative study exploring the barriers and facilitators to ART administration amongst children younger than five years. Children were divided into three groups based on their VL at the time of recruitment. Children on ART for ≥1 years with a VL ≤400 copies per millilitre (cps/ml) were categorised as virally suppressed. A suppressed VL was considered an indicator of good adherence. Children on ART ≥1 year with two consecutive VLs of ≥400 cps/ml were categorised as virally unsuppressed. An unsuppressed VL was considered to be an indicator of poor adherence. Children on ART ≤1 year with no VL data were “new enrollers”. The inclusion of new enrollers provided an early indication of how well caregivers understood their child’s illness and treatment.

### Procedure

The adherence counsellor at each clinic was asked to invite caregivers attending the clinic on the child’s behalf to meet with the researchers in a private room after their clinic visit. We explained the objectives of the study to participants in their first language, isiZulu. Once a caregiver and child had been recruited, we accompanied them to a counselling session on the child’s next clinic date. Caregivers who were not present at their next clinic date were telephoned the following day to determine the reason. Adherence counsellors at each clinic and caregivers provided written informed consent for observations to take place.

### Ethics

The study received ethical approval from Stellenbosch University (S12/05/135), reciprocity with the University of KwaZulu-Natal (KZN), permission from the local Community Advisory Board and the hospital management.

## Data analysis

### Counselling data

The source of the counselling sessions’ data was the written notes of the principal investigator and research assistant’s observations of these sessions. The observations were structured using a semi-structured observation schedule, which elicited the following information: the length of the counselling session, presence of the child in the session, and content of the counselling session. Thus, the average length of the counselling observations and whether the child attended the counselling session or not were calculated using statistical functions in Excel 2013. Textual data regarding the content addressed in the counselling sessions were coded using ATLAS.ti v7. The counselling sessions were mostly conducted in isiZulu, and captured via the research assistant’s field notes. These field notes were translated into English before analysis.

## Results

### Sample characteristics-children

Our final sample consisted of 25 caregiver–child dyads. Thus, we observed 6 out of 10 of the newly initiated children, 11 out of 12 of the suppressed children, and 8 out of 11 of the unsuppressed children. Of the eight counselling sessions that did not take place, four caregivers stated that they were unwilling to wait in the long queues, three caregivers stated that they were unable to get time away from work, and one stated that she was in school and unable to attend. Children were mostly female (17 out of 25), with ages ranging from 2 to 5 years old. The primary caregiver to most of the children (15 out of 25) was their biological mother. Other primary caregivers included grandmothers (7 out of 25), 1 biological father, and 2 aunts.

### Sample characteristics – caregivers

The ages of the caregivers ranged from 22 to 67 years. All caregivers were isiZulu speakers and the majority were single and resided in households with other adults and children. Nine caregivers attended but did not complete Grade 12. The majority of caregivers were unemployed and had a household income of less than R12,000 (£518. 65) per month. A large proportion of the caregivers were also on ART, and most of them had received the pre-ART adherence counselling sessions for their children’s treatment. Of the six who did not receive the pre-ART education sessions, one was a biological mother who was ill at the time of her child’s treatment initiation and thus her sister attended the classes on her behalf. The remaining five had become the primary caregiver to the child post-treatment initiation and had never received any education about ART (Table 1).

**Table 1.** Caregiver characteristics.

Variable	Suppressed (*n* = 11)	Unsuppressed (*n* = 8)	Newly initiated (*n* = 6)	Total (*N* = 25)
*Age*, years, median (IQR) (Range: 22–67)	35 (27.5–48)	29 (24–40)	28.5 (25.5–51)	
*Marital status*				
Single	5	6	4	15
Married or living with a significant other in a permanent union	6	2	2	10
*Living arrangement*				
Live with children only	3	1	3	7
Live with other adults and children	8	7	3	18
*Education*				
No formal education	0	2	1	3
Completed primary school	2	3	2	7
Attended high school but did not complete Grade 12	5	2	2	9
Completed Grade 12	4	1	1	6
*Employment*				
Unemployed	9	8	4	21
Employed full-time	0	0	1	1
Student	1	0	1	2
Retired	1	0	0	1
*Income*				
Less than R12,000	5	6	0	11
Do not know	2	0	1	3
CSG	3	2	2	7
>1 grant	1	0	3	4
*Caregiver on ART*, yes, no	8	6	5	19
*Caregiver attend HIV education session*, yes	8	7	4	19

Note: CSG = Child Support Grant.

### Counselling observations

On average, counselling visits across all observations lasted 8.1 minutes. Only 10 of the counselling sessions lasted longer than 10 minutes. However, six of the sessions were longer as a result of administrative tasks that took place during the sessions. For example, in one session, this was because both caregiver and child were counselled in the same session. The counsellor spent the counselling time conducting a pill count of the caregivers’ own medication (Table 3). In two sessions, the counsellor spent the counselling time replacing old file covers with new ones. In one session, counselling for two children (twins on ART) took place. In another session that lasted 18 minutes, 12 minutes was spent searching for the laboratory results of the child in the filing room next door to the counsellor’s room.

Children accompanied their caregiver to the counselling session in 18 out of 25 observations. Almost all (24 out of 25) caregivers who attended the counselling sessions were the primary caregiver of the child. In eight of the observations, the counselling sessions lacked privacy. In three instances, two counsellors occupied one counselling room and counselled their patients simultaneously. In other instances, counselling sessions were interrupted by other members of staff and in two instances, the counselling sessions were interrupted by other patients (Table 2).

**Table 2.** Counselling observations across the three criteria groups.

	Newly initiated	Suppressed VLs	Unsuppressed VLs	Total *N* = 25
Number of observations	6	11	8	25
Length of counselling session in minutes (mean, [range])	8.2 [5,10]	8.8 [4, 18]	6.86 [2, 14]	8.1
Presence of child in session	5	6	7	18
Presence of primary caregiver	5	11	8	24^a^
Caregiver and child counselled in same session, yes	1	1	1	3
Number of interruptions during counselling sessions^b^	1	2	2	5
>1 counselling session taking place, yes	1	0	2	3

^a^For one child (newly initiated on ART), an aunt attended the counselling session on the primary caregiver’s (mothers) behalf. In eight of the observations, the counselling sessions lacked privacy.

^b^Counselling sessions were interrupted by other members of staff and by other patients.

### Content addressed in the sessions

There were only two observed instances in which caregivers reported issues related to the child to the counsellor. In one instance, the child’s eyes were hurting and in the other, the caregiver reported sores on the child’s body. In both instances, the counsellors recommended that the caregiver take the child to see a doctor (Table 3).

**Table 3.** Counselling observations and activities across the three criteria groups.

	Newly initiated	Suppressed	Unsuppressed	Total
Content covered in session	Number of times occurred (out of 6 observations)	Number of times occurred (out of 11 observations)	Number of times occurred (out of 8 observations)	Out of 25 observations
*Physical issues*				
Caregiver reported child’s eyes are hurting			1	1
Caregiver reported sores on child’s body to counsellor	1			1
*Caregiver knowledge of treatment*				
Counsellor asked caregiver about dose amounts	5	3	2	10
Counsellor asked caregiver about dose times	1		2	3
Counsellor asked caregiver about medication names		1	2	3
Counsellor asked caregiver to demonstrate measurements	2			2
*Practicalities*				
Counsellor asked caregiver about number of treatment givers	1	1	1	3
Counsellor asked caregiver about reminder tools		1		1
Counsellor asked caregiver to bring child for blood tests	1	1	2	4
Counsellor asked caregiver to bring child to counselling	1	3		4
Counsellor asked caregiver to bring medication to clinic		1		1
*Reporting of lab results*				
Counsellor reported child’s CD4 count to caregiver		2	2	4
Counsellor reported child’s weight to caregiver	2	1	1	4
Counsellor reported child’s VL to caregiver		3	4	7
Counsellor did caregiver’s pill count	1	1		2
*Other*				
Counsellor searched for lab child’s results	1	1		2
Counsellor asked caregiver how child was doing		2		2
Counsellor asked the age of the child			1	1

During the counselling sessions, counsellors typically asked about the dose amounts of medication administered to the child (10 out of 25 observations), reported the child’s weight to the caregiver (4 out of 25 observations), reported the child’s CD4 count (4 out of 25 observations) and VL results to the caregiver (7 out of 25 observations), and in one instance, a counsellor asked about the use of a reminder tool for medication times (e.g., a mobile phone alarm).

In four observations, counsellors reported the VL status of an unsuppressed child to the caregiver. In two of these observations, the counsellors stated that the child was due to have their VLs tested again at the next clinic visit. In the other two sessions, the counselling lasted less than 10 minutes and counsellors asked caregivers to name the medications the child was receiving and to name the dose amounts. Both counsellors asked what times the caregivers were administering the medications to the child and emphasised that the timing was important. In both the above instances, the caregiver of the child was their grandmother. Both caregivers were able to name the dose amounts. However, one caregiver was unable to name all of the medications and reported that she shared the responsibility of clinic attendance and treatment administration with two of the child’s older siblings. Irregular clinic attendance by the child’s grandmother may have contributed to her inability to name all of the medications the child received. In neither of these sessions did counsellors explore the difficulties the caregivers were having with treatment preparation or administration.

There were two instances (both children newly initiated on ART), in which the counsellors had asked the caregivers to demonstrate how they measured the medications. In both instances, the counsellor had presented the caregiver with a syringe and cup of water and asked the caregiver to demonstrate how much of a certain formulation (in both cases Lopinavir/ritonavir) the caregiver administered to the child. Both caregivers had made accurate measurements. There were two counselling sessions (both unsuppressed children on ART) in which the counsellor attending to the caregivers had only signed the patients’ files for attendance and had no discussion at all about the patients’ treatment.

## Discussion

The IMB model emphasises that accurate information, sufficient motivation and behavioural skills are all necessary requirements to improve adherence to ART. Our data show that caregivers attending a public healthcare facility in rural South Africa do not receive adherence counselling consistent with these requirements. Lay counsellors typically receive little support and supervision beyond their initial training which places them at a relative disadvantage to engage with patients in a supportive manner that may improve patients’ adherence to ART (Coetzee et al., 2015; Dewing et al., 2012, 2015; Kagee, 2013; Petersen et al., 2014; Remien et al., 2013). As such, we contextualise poor adherence counselling as a consequence of an overburdened healthcare system that lacks sufficient support structures for both the staff and the ART user.

As seen from the results, counselling sessions took place within a very short amount of time. Furthermore, counsellors were often involved in administrative tasks during counselling sessions. Given the various roles of lay health personnel in the South African healthcare system, it is necessary to allocate specific roles to each of the health workers. For example, some of the counsellors could specifically be involved with sorting of patient files and filing lab results, while other counsellors could focus specifically on the one-on-one counselling with patients. The organisation of roles will provide counsellors with a renewed purpose in their role. Evidence shows that counsellors can be trained to provide psychosocial support and adherence counselling to ART users (Petersen et al., 2014). However, in the absence of regular follow-up training and supervision, it is apparent that the intervention sessions provide little sustainable use. Providing adherence counsellors with regular support and debriefing is likely to improve morale, which has shown to facilitate HIV care provision to patients (Kagee, 2013).

Lay counsellors are often members living and residing in the same communities as their patients. Thus, they are continually confronted with barriers that may leave them feeling helpless in terms of offering solutions to their patients’ problems. When continually faced with issues that are beyond the scope of their ability, it is no wonder that counsellors may resist probing for difficulties in the lives of their patients.

## Conclusions

We have shown that the level of adherence counselling that patients receive is sub-optimal and the content, quite minimal. Inadequate counselling services are most likely due to poor training of counsellors, which requires further study. The training that counsellors receive should prioritise skills to continually monitor caregivers’ knowledge about ART, motivation to adhere, and skills in administering treatment to children. Satisfying only one of the conditions will not be sufficient for optimal adherence to the regimen. The adherence counselling sessions provide both the space and time for such practices to be monitored. There are structural issues at play also. Counselling rooms often lack space and privacy, which is a consequence of an overburdened healthcare system. Structural interventions such as creating suitable spaces for counselling sessions also need to be prioritised. Unless these issues are addressed, the numbers of children with raised VLs and resistance to antiretroviral drugs will continue to rise.
